# Oxidative stress in critically ill neonatal foals

**DOI:** 10.1111/jvim.17297

**Published:** 2025-01-24

**Authors:** David Wong, Dipak Kumar Sahoo, Cosette Faivre, Jamie Kopper, Katie Dersh, Theresa Beachler, Melissa Esser

**Affiliations:** ^1^ Department of Veterinary Clinical Sciences, College of Veterinary Medicine Iowa State University Ames Iowa USA

**Keywords:** antioxidant, ascorbic acid, oxidative injury, reactive oxygen species, sepsis

## Abstract

**Background:**

Oxidative injury occurs in septic people, but the role of oxidative stress and antioxidants has rarely been evaluated in foals.

**Objectives/Hypothesis:**

To measure reactive oxygen species (ROS), biomarkers of oxidative injury, and antioxidants in neonatal foals. We hypothesized that ill foals would have higher blood concentrations of ROS and biomarkers of oxidative injury and lower concentrations of antioxidants compared to healthy foals.

**Animals:**

Seventy‐two hospitalized and 21 healthy neonatal foals.

**Methods:**

Prospective cohort study. Reactive oxygen species (hydrogen peroxide [H_2_O_2_]), biomarkers of oxidative injury (malondialdehyde [MDA], protein carbonyl), and antioxidants (superoxide dismutase [SOD], catalase [CAT], glutathione, and glutathione reductase [GR] and peroxidase [GPx]) were measured from foals at admission. Measured variables were compared between healthy and ill foals using a 1‐way ANOVA by Tukey's multiple comparisons test.

**Results:**

Ill foals (n = 51) had significantly higher mean concentrations of H_2_O_2_ (healthy 2.6 ± 1.4 nmol/mL, ill 6.8 ± 4.6 L nmol/mL; 95% CI), MDA (healthy 31.2 ± 14.4 nmol/mL, ill 114.3 ± 94.0 nmol/mL; 95% CI), and protein carbonyl (healthy 0.07 ± 0.01 nmol/mg protein, ill 0.12 ± 0.02 nmol/mg protein, 95% CI). Significant lower CAT (healthy 0.4 ± 0.3 mU/mg protein, ill 0.02 ± 0.02 mU/mg protein, 95% CI), glutathione (healthy 238.5 ± 101.9 μg/mL, ill 110.7 ± 37.8 μg/mL, 95% CI; *P* < .0001), GR (healthy 1.6 ± 1.8 mU/mg protein, ill 0.4 ± 0.5 mU/mg protein, 95% CI), and GPx (healthy 0.01 ± 0.003 mU/mg protein, ill 0.007 ± 0.002 mU/mg protein, 95% CI) were also noted.

**Conclusions and Clinical Importance:**

Oxidative stress and lower antioxidant concentrations occur in ill and bacteremic neonatal foals. These variables should be considered during the treatment of ill foals.

**Abbreviations**
CAT: catalase
d‐ROMs: derivatives of reactive oxygen metabolites
GPx: glutathione peroxidase
GR: glutathione reductase
GSH : GSSG: reduced to oxidized glutathione
H_2_O_2_
: hydrogen peroxide
MDA: malondialdehyde
PUFA: polyunsaturated fatty acids
ROS: reactive oxygen species
SOD: superoxide dismutase
SOFA: sequential organ failure assessment
TBARS: thiobarbituric acid reactive substances

## 1 INTRODUCTION

Sepsis is a common problem in neonatal foals.^1^, ^2^, ^3^ The invasion of tissues by pathogens causes numerous downstream processes resulting in the clinical syndrome of sepsis. Some of these processes include inflammatory and oxidative mechanisms that can act independently from the presence of the pathogens themselves.^4^, ^5^ During sepsis, the inflammatory and oxidative responses aid in containment of infection, participate in bacterial clearance, and facilitate reparative processes.^5^, ^6^ However, exuberant host responses to infection contribute to tissue damage, organ dysfunction, and death. The body attempts to control inflammatory and oxidant injury by maintaining endogenous anti‐inflammatory and antioxidant systems.^7^ However, the anti‐inflammatory response is frequently overwhelmed in sepsis, leading to the systemic inflammatory response syndrome (SIRS). Additionally, antioxidant responses can be exhausted, resulting in elevated oxidative burden, redox imbalance favoring oxidative pathways, and oxidative stress.^6^, ^8^

Oxidative stress is an imbalance between oxidants and antioxidants at the cellular level that results in oxidative modification of cellular macromolecules (apoptosis, necrosis) and structural tissue damage.^9^, ^10^ Activation of prooxidant pathways and production of reactive oxygen (ROS) and nitrogen species is well documented in septic infants along with an increase in antioxidant activity.^4^, ^6^, ^8^ However, antioxidant activity is not always able to compensate for increased oxidative burden resulting in detrimental cellular effects documented by increased markers of oxidative damage.^11^, ^12^, ^13^ Septic newborn infants have significantly higher concentrations of malondialdehyde (MDA; a marker of lipid peroxidation) along with decreased antioxidant enzymes such as superoxide dismutase (SOD), catalase (CAT), and glutathione peroxidase (GPx) when compared to healthy controls.^14^, ^15^, ^16^, ^17^

In contrast to the vast amount of research related to sepsis‐induced oxidative injury in people, there is minimal knowledge in foals. In 1 study, ill foals did not have a significant degree of oxidative stress as determined by measurement of 3‐nitrotyrosine (3‐NT).^18^ Antioxidant molecules such as serum selenium concentrations were lower in ill foals, but no difference was detected in serum vitamin E or GPx concentrations between groups.^18^ Concentrations of ascorbic acid are also lower in septic and ill foals compared to healthy foals.^19^

Because of the lack of sufficient information regarding the role of oxidative injury in ill and septic foals, the objectives of this study were to determine if there are differences in ROS (hydrogen peroxide [H_2_O_2_]), oxidative stress markers (MDA, protein carbonyl), and antioxidant molecules (SOD, CAT, glutathione, GPx, glutathione reductase [GR]) between healthy, ill, and bacteremic neonatal foals. The hypothesis was that ill and bacteremic foals would have elevated ROS and markers of oxidative stress and decreased concentrations of antioxidant molecules compared to healthy foals.

## 2 MATERIALS AND METHODS

### 2.1 Animals

#### 2.1.1 Healthy foals

Twenty‐one foals born at the university horse farm were examined within 24 hours of birth. Foals were considered healthy if the foals had a normal physical examination, vital signs within reference intervals, reached specific benchmarks (eg, standing, ambulation, nursing) within 2 hours, and had a serum IgG concentration >800 mg/dL measured at ≤24 hours of age.

#### 2.1.2 Hospitalized foals

Seventy‐two foals presented to, or born at, the Lloyd Veterinary Medical Center between January and July 2023 were included. All foals were <30 days of age, with 13 foals born at the hospital. Newborn foals were categorized as nonseptic hospitalized controls if these foals met the criteria noted for healthy university‐owned foals and had an updated sepsis score of ≤5.^20^ All historical, clinicopathologic, and physical examination information necessary to complete the updated sepsis score was available for each foal to be included in the study.^20^ All hospitalized foals had blood collected for culture as described below. Hospitalized foals were grouped via 3 methods: Method 1: blood culture‐positive and blood culture‐negative categories; Method 2: stratified into illness groups based on individual updated sepsis score^20^ and categorized as hospitalized controls (sepsis score 0‐5), mild (sepsis score 6‐11), moderate (sepsis score 12‐17), or severe (sepsis score 18‐29) illness. Method 3: All ill foals (sepsis score 6‐29).

### 2.2 Study design

This study was approved by the Animal Care and Use Committee (#22‐228) and was a single‐center, prospective study involving privately‐ and university‐owned foals. In healthy university‐owned foals, 15 mL of blood was collected by venipuncture within 24 hours of birth. Samples were split between EDTA and clot tubes. Within 30 minutes, EDTA and clot tubes were centrifuged for 5 minutes, and plasma and serum, respectively, were harvested and placed in polypropylene tubes and frozen at −80°C.

In privately owned hospitalized foals, blood (35 mL) was collected at admission either by venipuncture or immediately after placement of a jugular vein catheter. If the foal was born at the hospital, blood was drawn between 12 and 18 hours of age. Two blood culture bottles (aerobic and anaerobic) were inoculated, each with 10 mL of blood for culture; blood cultures were performed using routine methods with microorganisms identified using standard identification techniques. The remaining blood was split between EDTA and clot tubes that were processed as above and frozen at −80°C.

At the end of the sample collection period, the following variables were measured from blood samples from each foal: hydrogen peroxide (H_2_O_2_), MDA, protein carbonyl, SOD, CAT, glutathione (total, oxidized, reduced), GR, and GPx.

### 2.3 Measurement of markers of oxidative stress and antioxidant activity

Assays used in this study (H_2_O_2_, MDA, protein carbonyl, SOD, CAT, glutathione, GR, GPx) were purchased from Abcam (Abcam, Waltham, MA) and performed according to manufacturer's instructions. Please see Supporting Information (File S1) for complete methods and assay details.

### 2.4 Statistical analysis

The data among different groups were compared using 1‐way ANOVA followed by Tukey's multiple comparisons test by GraphPad Prism 9 (https://graphpad.com/). Minimal statistical significance was accepted at *P* < .05.

## 3 RESULTS

This study consisted of 21 healthy university‐owned control foals (≤24 hours of age) and 72 client‐owned hospitalized foals. Of the 72 client‐owned foals, the average age at presentation was 41 hours (range, 0‐288 hours) and consisted of 38 colts and 34 fillies. Breeds represented included Quarter Horse (n = 25), Thoroughbred (18), Paint (5), Belgian (4), Standardbred (4), Percheron (3), Warmblood (2), Tennessee Walker (2), Pony of America (2), Friesian (2), and 1 of each of the following: Clydesdale, Hackney Pony, Gypsy Vanner, Morgan, and Miniature Horse.

Of the 72 hospitalized foals, 35 of 72 foals (48.6%) had positive bacterial growth with blood culture. The median (range) updated sepsis score of the 72 hospitalized foals was 7.5 (range, 0‐29); the median (range) updated sepsis score for blood culture positive and negative foals was 11.5 (5‐29) and 10 (6‐20), respectively. Hospitalized foals were divided into illness categories (Method 2) based on the updated sepsis score (Table 1) with 23 in the hospital control category and 21, 16, and 12 in the mild, moderate, and severe illness category, respectively. Collectively, there was a total of 51 ill foals (Method 3) with a median updated sepsis score of 11 (range, 5‐29). Of the hospitalized foals, 60 survived to discharge and 12 died or were euthanized because of a grave prognosis (overall survival rate 83%).

**TABLE 1:** Characteristics of hospitalized foals stratified into illness groups based on updated sepsis score.

Category	Sepsis score median (range)	Number of foals in category	Number of blood culture positive	Survival to discharge	SIRS positive	Blood L‐lactate (mmol/L)
Hospital control	4 (0‐5)	23	2/23 (9%)	22/23 (96%)	0/23 (0%)	1.9 ± 1.1
Mild	7 (5‐10)	21	14/21 (67%)	19/21 (91%)	1/21 (5%)	2.8 ± 1.8
Moderate	12.5 (11‐17)	16	8/16 (50%)	12/16 (75%)	8/16 (50%)	6.6 ± 4.7
Severe	18 (5‐29)	12	11/12 (92%)	7/12 (58%)	10/12 (83%)	9.1 ± 6.2

*Note*: Foals were categorized as hospitalized controls (sepsis score 0‐5) and mild (sepsis score 6‐11), moderate (sepsis score 12‐17), or severe (sepsis score 18‐29) illness.

### 3.1 Comparison of various oxidative stress and antioxidant variables

**FIGURE 1:**
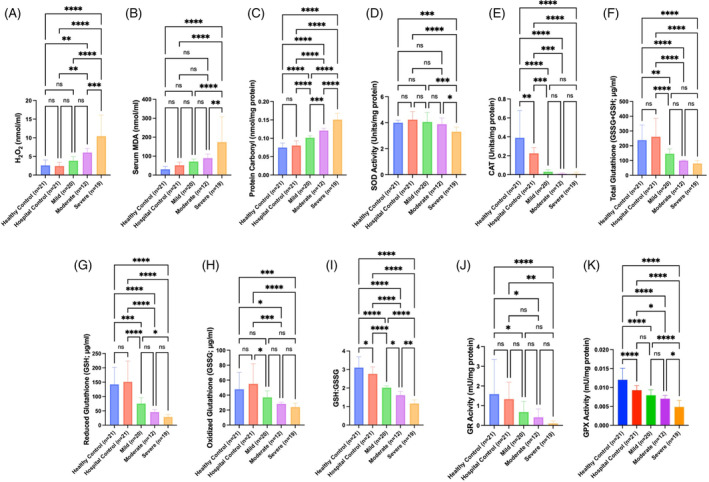
(A‐K) Comparison of various measured variables between control groups and mild, moderate, and severe illness groups. *Indicates a statistically significant difference between groups; please see the results section for specific *P* values. CAT, catalase; GPX, glutathione peroxidase; GR, glutathione reductase; GSH : GSSG, reduced to oxidized glutathione ratio; H_2_O_2_, hydrogen peroxide; MDA, malondialdehyde; ns, not significant; SOD, superoxide dismutase.

A summary of the values of various analytes from different groups of foals is documented in Figure 1 and Table 2.

**TABLE 2:** Mean ± SD values of various analytes from neonatal foals in different control and illness groups.

	Healthy control	Hospital control	Mild	Moderate	Severe	Blood culture +	Blood culture −	Survival	Nonsurvival
Analyte	(n = 23)	(n = 21)	(n = 21)	(n = 16)	(n = 12)	(n = 35)	(n = 37)	(n = 60)	(n = 12)
H_2_O_2_ (nmol/mL)	2.60 ± 1.44	2.42 ± 0.98	3.86 ± 1.10	6.06^a^, ^b^ ± 1.06	10.44^a^, ^b^, ^c^, ^d^ ± 5.65	7.78^a^, ^b^, ^e^ ± 4.80	4.06 ± 2.15	5.95^f^ ± 3.2	9.68 ± 6.93
MDA (nmol/mL)	31.2 ± 14.4	52.3 ± 16.1	71.9 ± 12.9	89.7 ± 21.6	174.4^a^, ^b^, ^c^, ^d^ ± 133.6	130.6^a^, ^b^, ^e^ ± 103.9	66.6 ± 14.3	86.0^f^ ± 24.5	206.1 ± 161.4
PC	0.07 ± 0.01	0.08 ± 0.01	0.10^a^, ^b^ ± 0.01	0.12^a^, ^b^, ^c^ ± 0.01	0.15^a^, ^b^, ^c^, ^d^ ± 0.02	0.13^a^, ^b^, ^e^ ± 0.03	0.11^a^, ^b^ ± 0.01	0.11^f^ ± 0.02	0.16 ± 0.02
SOD	4.00 ± 0.19	4.24 ± 0.62	4.06 ± 0.72	3.89 ± 0.47	3.31^a^, ^b^, ^c^, ^d^ ± 0.36	3.53^a^, ^b^, ^e^ ± 0.37	4.36 ± 0.85	3.90^f^ ± 0.62	3.23 ± 0.39
CAT	0.39 ± 0.29	0.23^a^ ± 0.06	0.03^a^, ^b^ ± 0.02	0.01^a^, ^b^ ± 0.01	0.01^a^, ^b^ ± 0.02	0.01^a^, ^b^ ± 0.02	0.03^a^, ^b^ ± 0.02	0.02 ± 0.02	0.01 ± 0.02
Total GSH (μg/mL)	228.5 ± 101.9	261.4 ± 124.7	146.2^a^, ^b^ ± 32.4	99.7^a^, ^b^ ± 3.4	80.1^a^, ^b^ ± 19.5	99.03^a^, ^b^ ± 28.7	144.6^a^, ^b^ ± 41.5	120.9 ± 35.7	77.2 ± 21.6
GSH (μg/mL)	142.6 ± 59.4	151.4 ± 72.5	75.5^a^, ^b^ ± 20.6	45.9^a^, ^b^ ± 8.1	28.7^a^, ^b^, ^c^ ± 8.6	42.39^a^, ^b^ ± 18.6	76.70^a^, ^b^ ± 25.31	58.6 ± 23.9	26.8 ± 9.3
GSSG (μg/mL)	47.9 ± 22.5	55.0 ± 26.8	37.0^b^ ± 8.7	28.3^a^, ^b^ ± 2.3	24.2^a^, ^b^ ± 5.1	27.19^a^, ^b^ ± 6.1	38.90^a^, ^b^ ± 8.8	32.6 ± 8.0	22.4 ± 4.5
GSH : GSSG	3.11 ± 0.58	2.77^a^ ± 0.38	2.02^a^, ^b^ ± 0.10	1.62^a^, ^b^, ^c^ ± 0.20	1.17^a^, ^b^, ^c^, ^d^ ± 0.19	1.50^a^, ^b^, ^e^ ± 0.39	1.93^a^, ^b^ ± 0.27	1.74^f^ ± 0.35	1.17 ± 0.24
GR	1.59 ± 1.76	1.34 ± 0.86	0.68^a^ ± 0.54	0.41^a^ ± 0.42	0.10^a^, ^b^ ± 0.12	0.21^a^, ^b^ ± 0.21	0.97^a^, ^b^ ± 0.56	0.51 ± 0.48	0.05 ± 0.11
GPx	0.0120 ± 0.0031	0.0093^a^ ± 0.0012	0.0079^a^ ± 0.0014	0.0070^a^, ^b^ ± 0.0009	0.0049^a^, ^b^, ^c^, ^d^ ± 0.0017	0.0062^a^, ^b^ ± 0.0019	0.0079^a^, ^b^ ± 0.0015	0.0073^f^ ± 0.0014	0.0045 ± 0.002

*Note*: Catalase, glutathione reductase, and glutathione peroxidase activities were expressed as mU/mg protein, superoxide dismutase as units/mg protein, and protein carbonyl contents were expressed as nmol/mg protein.

^a^ Significantly different than healthy control group.

^b^ Significantly different than hospital control group.

^c^ Significantly different than mild group.

^d^ Significantly different than moderate group.

^e^ Significantly different than blood culture‐negative group.

^f^ Significantly different than nonsurvival group.

#### 3.1.1 Hydrogen peroxide

**FIGURE 2:**
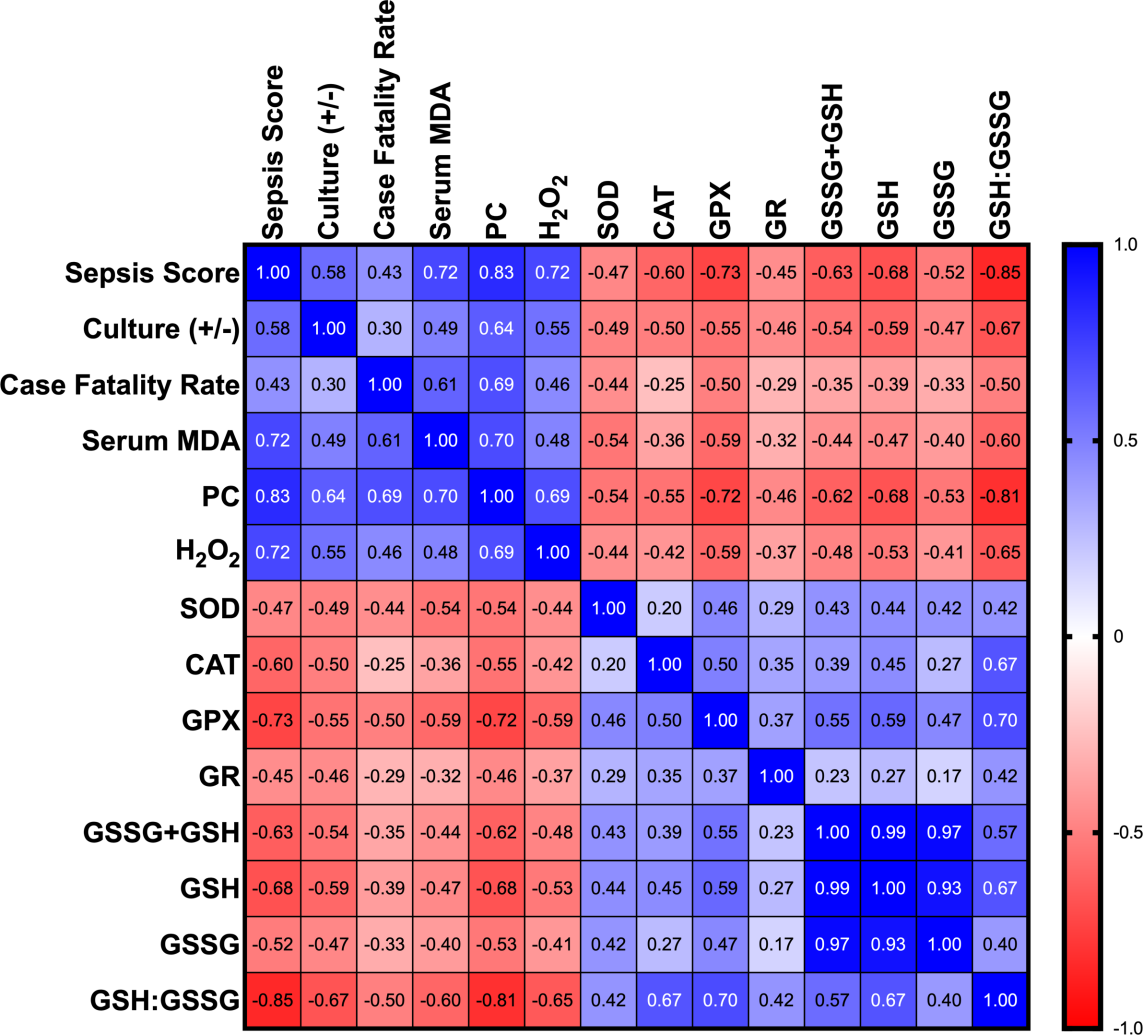
Heat map displaying Pearson correlation coefficients between different oxidative stress and antioxidant defense variables, culture status, sepsis score, and case fatality rate. Minimal statistical significance was accepted at *P* < .05. Positive correlations are in blue, and negative correlations are in red. CAT, catalase; culture (+/−), blood culture positive or negative; GR, glutathione reductase; GSH + GSSG, total glutathione; GSH, reduced glutathione; GSH : GSSG, reduced to oxidized glutathione ratio; GSSG, oxidized glutathione; H_2_O_2_, hydrogen peroxide; MDA, malondialdehyde; PC, protein carbonyl; SOD, superoxide dismutase.

There was no statistically significant difference in mean serum H_2_O_2_ concentrations between healthy control and hospital control groups (Figure 1A). Mean serum H_2_O_2_ concentrations (Table 2) were significantly higher in foals in the moderate (adjusted *P* < .007) and severe (adjusted *P* < .0001) illness groups compared to the control groups and was also significantly higher when the severe illness group was compared to the moderate (adjusted *P* = .004) and mild illness (adjusted *P* < .0001) groups. H_2_O_2_ concentrations were positively correlated with the updated sepsis score (*r* = 0.72; Figure 2). H_2_O_2_ concentrations were significantly higher in blood culture‐positive compared to blood culture‐negative foals (adjusted *P* = .004) and blood culture‐positive foals had significantly higher H_2_O_2_ concentrations when compared to control groups (adjusted *P* < .0001).

#### 3.1.2 Malondialdehyde

There was no statistically significant difference in mean serum MDA concentrations between healthy control and hospital control groups (Figure 1B). Serum MDA concentrations were significantly higher in the severe illness group compared to control groups (adjusted *P* < .0001); the severe illness group was significantly higher than mild (adjusted *P* < .0001) and moderate illness groups (adjusted *P* = .003, Table 2). Malondialdehyde concentrations were positively correlated with the updated sepsis score (*r* = 0.72; Figure 2). Serum MDA concentrations were significantly higher in blood culture‐positive compared to blood culture‐negative foals (adjusted *P* = .02) and blood culture‐positive foals had significantly higher serum MDA concentrations compared to control groups (adjusted *P* ≤ .003).

#### 3.1.3 Protein carbonyl

There was not a statistically significant difference in mean protein carbonyl concentrations between healthy control and hospital control groups (Figure 1C). Protein carbonyl concentrations (Table 2) were significantly higher in foals in the mild, moderate, and severe illness groups (adjusted *P* < .0001) compared to control groups. Protein carbonyl concentrations were significantly higher in the severe illness group compared to the moderate and mild illness groups (adjusted *P* < .0001), and the moderate illness group was significantly higher than the mild illness group (adjusted *P* = .0001). Protein carbonyl concentrations were positively correlated with the updated sepsis score (*r* = 0.83; Figure 2) and were also significantly higher in blood culture‐positive when compared to blood culture‐negative foals (adjusted *P* = .008). Both blood culture positive and negative groups had significantly higher concentrations of protein carbonyl compared to control groups (adjusted *P* ≤ .002).

#### 3.1.4 Superoxide dismutase

There was not a statistically significant difference in SOD expression between healthy control and hospital control groups (Figure 1D). SOD activity (Table 2) was significantly lower in foals in the severe illness group compared to the healthy (adjusted *P* = .004) and hospital control (adjusted *P* < .0001) groups. SOD was significantly lower in the severe illness group when compared to the mild (adjusted *P* = .0001) and moderate (adjusted *P* = .02) illness groups. SOD activity was significantly lower in blood culture‐positive compared to blood culture‐negative foals (adjusted *P* < .0001) and was significantly lower when blood culture‐positive foals were compared to control groups (adjusted *P* ≤ .004).

#### 3.1.5 Catalase

When compared to the healthy control group, mean serum CAT concentration was significantly lower in the hospital control group (adjusted *P* = .003, Figure 1E) as well as the mild, moderate, and severe illness groups (adjusted *P* < .0001). Serum CAT concentration (Table 2) was also significantly lower when the hospital control group was compared to the mild, moderate, and severe illness groups (adjusted *P* ≤ .0005). Catalase concentrations were negatively correlated with the updated sepsis score (*r* = −0.60; Figure 2). Both blood culture‐positive and culture‐negative groups had significantly lower catalase concentrations when compared to control groups (adjusted *P* ≤ .0008).

#### 3.1.6 Total glutathione

There was no statistically significant difference between healthy control and hospital control groups when evaluating mean serum total glutathione concentration (Figure 1F). Total glutathione concentrations (Table 2) were significantly lower in foals in the mild, moderate, and severe illness groups compared to the healthy (adjusted *P* ≤ .003) and hospital (adjusted *P* < .0001) control groups. Total glutathione concentrations were negatively correlated with the updated sepsis score (*r* = −0.63; Figure 2). Both blood culture‐positive and culture‐negative groups had significantly lower total glutathione concentrations when compared to control groups (adjusted *P* < .0001).

#### 3.1.7 Reduced glutathione

There was no statistically significant difference between the healthy control and hospital control groups when evaluating mean reduced glutathione concentrations (Figure 1G). Reduced glutathione concentrations (Table 2) were significantly lower in foals in mild, moderate, and severe illness groups when compared to control groups (adjusted *P* < .0001). Reduced glutathione was also significantly lower in the severe illness group when compared to the mild illness group (adjusted *P* = .02). Reduced glutathione concentrations were negatively correlated with the updated sepsis score (*r* = −0.68; Figure 2). Both blood culture‐positive and culture‐negative groups had significantly lower reduced glutathione concentrations compared to control groups (adjusted *P* ≤ .008).

#### 3.1.8 Oxidized glutathione

There was no statistically significant difference between healthy control and hospital control groups when evaluating mean oxidized glutathione concentrations (Figure 1H). Oxidized glutathione concentration (Table 2) was significantly lower in foals in moderate (adjusted *P* = .02) and severe (adjusted *P* = .0004) illness groups when compared to the healthy control group and significantly lower in mild (adjusted *P* = .01), moderate (adjusted *P* = .0005), and severe (adjusted *P* < .0001) illness groups compared to the hospital control groups. Both blood culture‐positive and culture‐negative groups were significantly lower than the control groups (adjusted *P* ≤ .0002).

#### 3.1.9 Ratio of reduced to oxidized glutathione (GSH : GSSG)

The hospital control group had a significantly lower GSH : GSSG ratio compared to the healthy control group (adjusted *P* = .02, Figure 1I). The GSH : GSSG ratio (Table 2) was also significantly lower in mild, moderate, and severe illness groups compared to control groups (adjusted *P* < .00001). The ratio was significantly lower in the severe illness group when compared to moderate (adjusted *P* = .008) and mild illness groups (adjusted *P* < .00001) and the moderate illness group was significantly lower compared to the mild illness group (adjusted *P* = .02). The GSH : GSSG ratio was negatively correlated with the updated sepsis score (*r* = −0.85; Figure 2). When comparing foals based on blood culture status, the GSH : GSSG ratio was significantly lower in the blood culture‐positive group when compared to the culture‐negative group; both groups were significantly lower than control groups (adjusted *P* < .00001).

#### 3.1.10 Glutathione reductase

There was no statistically significant difference between healthy control and hospital control groups when evaluating mean GR concentration (Figure 1J). Glutathione reductase concentration (Table 2) was significantly lower in foals in mild (adjusted *P* = .03), moderate (adjusted *P* = .01), and severe (adjusted *P* < .00001) illness groups compared to the healthy control group and was significantly lower in the severe illness group when compared to the hospital control group (adjusted *P* = .001). Both blood culture‐positive and culture‐negative groups were significantly lower compared to control groups (adjusted *P* ≤ .0002).

#### 3.1.11 Glutathione peroxidase

When compared to the healthy control group, mean GPx activity was significantly lower in foals in the hospital control group (adjusted *P* = .003, Figure 1K) and the mild, moderate, and severe illness groups (adjusted *P* < .0001). Serum GPx activity (Table 2) was also significantly lower when the hospital control group was compared to moderate and severe illness groups (adjusted *P* ≤ .0005) and the severe illness group was significantly lower than the mild (adjusted *P* < .0001) and moderate (adjusted *P* = .01) illness groups. Glutathione peroxidase concentrations were negatively correlated with the updated sepsis score (*r* = −0.73; Figure 2). Both blood culture‐positive and culture‐negative groups had significantly lower GPx activity when compared to the healthy control group (adjusted *P* ≤ .0001).

#### 3.1.12 Comparison of all ill foals to control groups

When all ill foals were compared (Method 3; n = 51) to healthy control and hospital control groups, significant increases in H_2_O_2_ (adjusted *P* < .0001, *P* < .0001, respectively), serum MDA (adjusted *P* < .0001, *P* = .003), and protein carbonyl (adjusted *P* < .0001, *P* < .0001) were observed along with significant decreases in total CAT (adjusted *P* < .0001, *P* < .0001), total glutathione (adjusted *P* < .0001, *P* < .0001), reduced (adjusted *P* < .0001, *P* < .0001) and oxidized (adjusted *P* < .0006, *P* < .0001) glutathione, GSH : GSSG (adjusted *P* < .0001, *P* < .0001), GR (adjusted *P* < .0001, *P* < .001), and GPx (adjusted *P* < .0001, *P* < .0001). Additionally, SOD was significantly lower (adjusted *P* = .003) in ill foals when compared to the hospital control group (Figure S1).

#### 3.1.13 Survival

Significantly higher mean concentrations of H_2_O_2_ (adjusted *P* = .005), MDA (adjusted *P* < .0001), and protein carbonyl (adjusted *P* < .0001), as well as significantly lower concentrations of SOD (adjusted *P* = .001), GSH : GSSG ratio (adjusted *P* = .0003), and GPx (adjusted *P* = .0002) were detected in ill foals that did not survive when compared to ill foals that survived to discharge.

## 4 DISCUSSION

Oxidative injury occurs in septic people, but the implications of oxidative stress and antioxidants remain inadequately investigated in ill foals. In the study presented here, ill and bacteremic foals had evidence of oxidative stress based on significantly higher blood concentrations of H_2_O_2_ and biomarkers of oxidative stress (MDA, protein carbonyl) along with significantly lower concentrations of several antioxidant enzymes (CAT, SOD, GR, GPx) and small antioxidant molecules (glutathione). Changes in these variables were directly (H_2_O_2_, oxidative stress biomarkers) or indirectly (antioxidant enzymes) correlated with illness severity (Figure 2). This information suggests that oxidative injury occurs in ill and bacteremic neonatal foals in the presence of lower concentrations of antioxidant enzymes. These findings are important as to help clinicians better understand the involvement of ROS, antioxidants, and cellular health in ill foals.

In this study, H_2_O_2_ was significantly elevated in moderate and severe illness groups when compared to control groups (Table 2) and was also significantly higher in the blood culture positive (eg, bacteremic) group when compared to the blood culture‐negative group. H_2_O_2_ is an unstable peroxide molecule that acts as a strong oxidant^21^, ^22^; under certain conditions, metal ions catalyze the cleavage of H_2_O_2_ and form hydroxyl radicals (eg, Fenton reaction), creating one of the most reactive radicals contributing to cell damage.^23^ In this study, the higher concentrations of H_2_O_2_ in foals based on illness score and positive blood culture suggest higher production of H_2_O_2_ or lower neutralization via endogenous antioxidants or a combination of these mechanisms in bacteremic foals with moderate to severe illness. Higher concentrations of H_2_O_2_ were also correlated with higher illness scores suggesting increased production of ROS with more severe illness.

Lipids (eg, polyunsaturated fatty acids [PUFA]), are prone to oxidative stress.^24^ Oxidants affect PUFA by extracting hydrogen, resulting in the formation of unstable lipid radicals. Subsequently, an oxygen molecule is inserted thereby generating lipid peroxyl radicals resulting in a chain reaction and formation of more stable compounds.^24^ During this process (lipid peroxidation), secondary products such as MDA are derived. In this study, MDA was significantly elevated in ill foals when compared to control groups and the mean MDA concentrations were significantly higher in more severe illness groups (Table 2). In addition, MDA concentration was significantly higher in blood culture‐positive compared to the blood culture‐negative group. Elevations in MDA in this study mimic human sepsis studies^14^, ^25^, ^26^, ^27^ and suggest that there is oxidative damage to cell membranes in bacteremic neonatal foals and this oxidative injury increases with worsening disease severity.

Protein carbonyl is formed by a variety of ROS through pathways such as direct oxidation of amino acids, oxidative cleavage of the protein backbone, or reaction of reactive aldehydes with amino acids.^26^ Protein carbonyl concentrations were significantly higher in foals in the mild, moderate, and severe illness groups when compared to control groups, with increasing concentrations positively correlated with increasing illness severity. Moreover, blood culture‐positive foals had significantly higher protein carbonyl concentrations when compared to blood culture‐negative foals. This suggests that there is damage to proteins in bacteremic foals and this injury increases with elevated illness scores.

Both enzymatic (SOD, CAT, GR, GPx) and nonenzymatic antioxidant systems (glutathione) are present to combat oxidants.^7^, ^9^, ^28^, ^29^, ^30^ In the study here, concentrations of SOD, CAT, GR, GPx, and glutathione were significantly lower in the ill foal groups when compared to control groups with a trend of progressively lower CAT concentrations with increasing illness scores. The findings in this study are similar to reports in people.^28^, ^31^ For example, 1 study documented significantly increased concentrations of oxidants while noting significantly lower concentrations of antioxidant enzymes (SOD, CAT) in septic human patients when compared to healthy controls.^28^ Moreover, SOD and CAT showed a negative correlation with the sequential organ failure assessment (SOFA) score^28^; in the foal study presented here, CAT was negatively correlated with the updated sepsis score.^28^ Although SOD concentrations were significantly lower in the blood culture‐positive group, no difference was detected in catalase or total glutathione between the blood culture‐positive and negative groups. Results from this study suggest that there are decreased antioxidants in ill foals, and some of these deficiencies (CAT) are negatively correlated with disease severity. These findings support previous reports that demonstrated a decrease in GPx activity during a murine sepsis model^5^, ^32^, ^33^ and a reduction in GPx (nonstatistically significant) in septic foals.^18^

Glutathione helps maintain a robust antioxidant system and has 2 forms, reduced (GSH) and oxidized (GSSG).^34^ Glutathione reductase maintains the cellular concentrations of GSH by reducing GSSG back to GSH.^35^ Glutathione (GSH) is a potent scavenger, with evidence suggesting that lower GSH concentrations are associated with the severity of pathological conditions, including sepsis.^5^, ^7^, ^11^, ^12^, ^36^, ^37^, ^38^, ^39^, ^40^ The balance between reduced glutathione (GSH) and oxidized glutathione (GSSG) is also an indicator of oxidative stress.^41^ The present study observed a decline in the serum GSH : GSSG ratio in foals with moderate to severe sepsis scores. Our study aligns with a previous study that demonstrated a decrease in tissue GSH concentrations following cecal ligation and puncture^42^, ^43^ and lower concentrations of glutathione in the blood of infected rats.^44^ This decrease in glutathione concentrations may be because of its consumption in sepsis‐induced oxidative stress.

A few studies have investigated antioxidants, plasma antioxidant capacity, and biomarkers of oxidative stress, such as derivatives of reactive oxygen metabolites (d‐ROMs) and thiobarbituric acid reactive substances (TBARS) in healthy foals.^45^, ^46^ One study documented elevated TBARS concentrations in healthy foals in the immediate postparturient period (5 minutes after birth) compared to later time points (12 and 168 hours of age) and suggested that there is an initial prooxidant balance during the early newborn period.^46^ The authors suggested that the higher concentrations of oxidants activate the antioxidant systems and help establish oxidative homeostasis during the first week of life.^46^ A different study noted that plasma antioxidant capacity increases over the first week of life in healthy foals.^45^ Thus, oxidative stress might be more prevalent in newborn foals for the proposed reason that the newborn is born into a comparatively hyperoxic extrauterine environment caused by increased oxygen bioavailability while still having an undeveloped antioxidant system that matures over time.^45^, ^47^

Measurement of antioxidant concentrations and biomarkers of oxidative stress have been examined in foals with pneumonia^48^, ^49^ and sepsis.^18^, ^50^ Foals with pneumonia have significantly higher oxidative stress biomarkers in blood (d‐ROMs, oxidative stress index) and breath condensate (H_2_O_2_) compared to healthy foals.^49^ Serum selenium and plasma GPx are significantly lower in severely ill foals compared to healthy foals; however, 3‐nitrotyrosine is significantly lower in severely ill foals when compared to controls suggesting that oxidative stress is lower in ill foals.^18^ The authors concluded that oxidative stress did not occur in septic foals, but this study involved only 8 severely ill foals.^18^ Another study examined TBARS in 129 sick neonatal foals, of which 29 were septic.^50^ In that study, no significant differences were detected in TBARS concentrations between septic and sick nonseptic foals or nonsurviving foals.^50^ The reason why the study presented here demonstrated significant elevations in biomarkers of oxidative stress and decreased antioxidants when compared to previously published studies is not known, but the timing of blood collection, percentage of septic foals in this study, severity of disease, and differences in biomarkers that were evaluated might contribute to variable results.

Several scoring systems are used in people to delineate the degree of illness, response to treatment, and predict survival; the SOFA score is widely used to evaluate the degree of organ dysfunction over time in ill patients.^51^, ^52^ The updated equine sepsis score was designed to identify foals with an increased likelihood of sepsis; however, in this study, it was also used to stratify severity of illness.^19^ While not the initial intent, this scoring system has many similarities with the SOFA score and helped determine severity of illness in this study. Both scoring systems evaluate organ function; however, the SOFA score has more specific criteria and cutoffs. For example, both systems evaluate kidney function via serum creatinine concentration; the SOFA score evaluates the cardiovascular system via mean arterial pressure and need for vasopressor therapy, whereas the updated sepsis score uses blood L‐lactate and heart rate. The coagulation system is evaluated via platelet count in the SOFA score, whereas the updated sepsis score uses presence or absence of petechia. The Glasgow coma score is used to evaluate the nervous system in the SOFA score, whereas the updated sepsis score uses findings of hypotonia, coma, lethargy, and seizures. The respiratory system is evaluated via the P_a_O_2_/F_i_O_2_ ratio in the SOFA score, whereas the presence of respiratory distress and respiratory rate are utilized within the sepsis score. Until a formal illness scoring system is developed and evaluated in foals, the authors suggest that the updated sepsis scoring system helps delineate the severity of illness.

Significant differences in several variables were detected between foals that survived compared to nonsurvivors. H_2_O_2_ was significantly higher in nonsurviving foals. Moreover, oxidative injury, as evidenced by significantly higher concentrations of MDA and protein carbonyl concentrations in nonsurviving foals, suggests that oxidative stress is elevated in severe illness and nonsurviving foals. In this study, only SOD and GSSG were significantly lower in nonsurviving foals suggesting that some antioxidants might be lower nonsurviving foals. However, the correlation between these variables and survival should be interpreted cautiously as most foals in the nonsurvival group were in extremis, but some foals might have survived with continued treatment. Furthermore, it is challenging to glean specific information regarding survival and oxidative stress as multiple factors contribute to the survival of ill neonatal foals.

Limitations of this study revolve around the specific antioxidants and biomarkers of oxidative injury that were measured. A wide range of variables were measured in this study, but it might have proven beneficial to measure other antioxidants such as ascorbic acid, vitamin E, and selenium as well as other biomarkers of oxidative stress.^53^ Practically, there are dozens of antioxidants and biomarkers of oxidative injury and therefore the authors chose to measure more common variables available. However, measurements of other biomarkers, including those that evaluate reactive nitrogen species, might have helped paint a clearer picture of the impact of reactive oxygen and nitrogen species and the redox state in ill foals. The lower sensitivity of a single blood culture (as opposed to multiple samples) to detect bacteremia is another limitation of this study.

In conclusion, the study presented here suggests that bacteremic and ill foals can be subject to oxidative injury based on elevations in H_2_O_2_ and biomarkers of cell membrane (MDA) and protein (protein carbonyl) damage; moreover, this study suggests that there are lower concentrations of some antioxidant enzymes (SOD, catalase, GR, GPx) based on significantly lower concentrations of these enzymes noted in ill foals. Whether or not the adjunctive administration of antioxidants to septic foals improves outcomes remains to be determined.

## CONFLICT OF INTEREST DECLARATION

Authors declare no conflict of interest.

## OFF‐LABEL ANTIMICROBIAL DECLARATION

Authors declare the off‐label use of ceftiofur, gentamicin, and amikacin which was administered to some foals enrolled in this study.

## INSTITUTIONAL ANIMAL CARE AND USE COMMITTEE (IACUC) OR OTHER APPROVAL DECLARATION

Approved by Iowa State University IACUC, protocol number 22‐228.

## HUMAN ETHICS APPROVAL DECLARATION

Authors declare human ethics approval was not needed for this study.

## Supporting information


**Figure S1.** Changes in antioxidant defense and oxidative stress parameters in neonatal foals from control and illness groups. The data are presented as mean ± SD values. The data among different groups were compared using 1‐way ANOVA followed by Tukey's multiple comparisons test by GraphPad Prism 9 (https://graphpad.com/). Minimal statistical significance was accepted at *P* < .05. Catalase, glutathione reductase, and glutathione peroxidase activities were expressed as mU/mg protein, superoxide dismutase as units/mg protein, and protein carbonyl contents were expressed as nmol/mg protein. Serum MDA and H_2_O_2_ concentrations were expressed as nmol/mL of serum sample. CAT, catalase; GPx, glutathione peroxidase; GR, glutathione reductase; GSH, reduced glutathione; GSSG, oxidized glutathione; H_2_O_2_, hydrogen peroxide; MDA, malondialdehyde; SOD, superoxide dismutase.

**File S1.** Complete methods and assay details.
