# Supplementary material for: Oxidative stress in critically ill neonatal foals
Source: J Vet Intern Med. 2025 Jan 24;39(1):e17297. doi: 10.1111/jvim.17297 (PMC11758150; doi:10.1111/jvim.17297)
Supplement: Supplementary file 1 — Figure S1. Changes in antioxidant defense and oxidative stress parameters in neonatal foals from control and illness groups. The data are presented as mean ± SD values. The data among different groups were compared using 1‐way ANOVA followed by Tukey's multiple comparisons test by GraphPad Prism 9 (https://graphpad.com/). Minimal statistical significance was accepted at P < .05. Catalase, glutathione reductase, and glutathione peroxidase activities were expressed as mU/mg protein, superoxide dismutase as units/mg protein, and protein carbonyl contents were expressed as nmol/mg protein. Serum MDA and H2O2 concentrations were expressed as nmol/mL of serum sample. CAT, catalase; GPx, glutathione peroxidase; GR, glutathione reductase; GSH, reduced glutathione; GSSG, oxidized glutathione; H2O2, hydrogen peroxide; MDA, malondialdehyde; SOD, superoxide dismutase. [file JVIM-39-e17297-s001.docx]

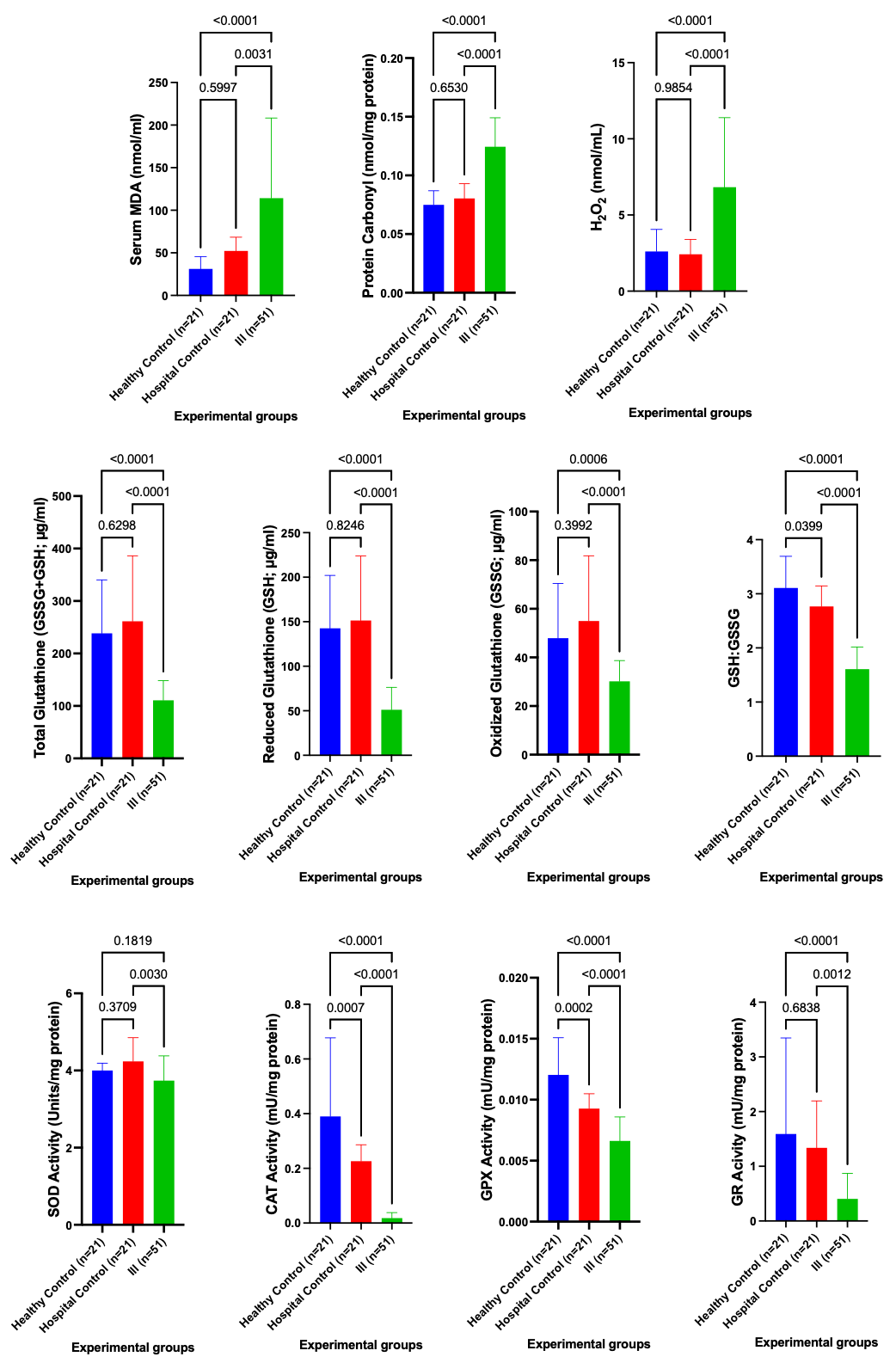


**Supplemental Figure S1.** Changes in antioxidant defense and oxidative stress parameters in neonatal foals from control and illness groups. The data are presented as Mean ± SD values. The data among different groups were compared using one-way ANOVA followed by Tukey’s multiple comparisons test by GraphPad Prism 9 (https://graphpad.com/). Minimal statistical significance was accepted at p<0.05. Catalase, glutathione reductase, and glutathione peroxidase activities were expressed as mU/mg protein, superoxide dismutase as units/mg protein, and protein carbonyl contents were expressed as nmol/mg protein. Serum MDA and H_2_O_2_ concentrations were expressed as nmol/mL of serum sample. H_2_O_2_, hydrogen peroxide; MDA, malondialdehyde; SOD, superoxide dismutase; CAT, catalase; GR, glutathione reductase; GSH, reduced glutathione; GSSG, oxidized glutathione; GPx, glutathione peroxidase.
