# Supplementary material for: Oxidative stress in critically ill neonatal foals
Source: J Vet Intern Med. 2025 Jan 24;39(1):e17297. doi: 10.1111/jvim.17297 (PMC11758150; doi:10.1111/jvim.17297)
Supplement: Supplementary file 2 — File S1. Complete methods and assay details. [file JVIM-39-e17297-s002.docx]

**Supplemental File: Materials & Methods**

**2.3 | Measurement of markers of oxidative stress** **and** **antioxidant activity**

All assays used in this study were purchased from Abcam (<https://www.abcam.com>; Abcam, Waltham, MA).

**Protein -** Serum and plasma protein concentrations were measured by using a bicinchoninic acid (BCA) protein assay kit. Briefly, 25 μl of each bovine serum albumin (BSA) standard and 25 times diluted serum and plasma protein samples were added into microtiter plate wells. Subsequently, 200 μl of BCA working reagent was added to the standard & sample wells, mixed, and then incubated at 37°C for 30 min. After incubation, the optical density (OD) 562 was recorded using SpectraMax M2e (Molecular Devices, San Jose, CA) microplate reader and the protein concentration (mg/mL) was calculated according to the manufacturer’s instructions.

**Hydrogen Peroxide (H_2_O_2_) –** To estimate serum H_2_O_2_, 50 μL of each serum sample or H_2_O_2_ standards (0.039 µM to 20 µM) were added to wells of a 96-well plate. Subsequently, 50 μL of catalyst was introduced into each well, mixed, and incubated for 5 minutes. A solution of 100 μL of dichlorodihydrofluorescin DiOxyQ (DCFH-DiOxyQ) was added to each well. The plate reaction wells were shielded from light and incubated for 30 minutes. The resulting fluorescence was measured using a SpectraMax M2e (Molecular Devices, San Jose, CA) fluorescence plate reader with an excitation wavelength of 480 nm and an emission wavelength of 530 nm. The H_2_O_2_ concentration (nmol/mL) was calculated according to manufacturer’s instructions.

**Lipid Peroxide -** Estimation of malondialdehyde (MDA) was performed by combining 20 μL of plasma with 500 μL of 42 mM H_2_SO_4_ in microcentrifuge tubes. Then, 125 μL of a solution containing phosphotungstic acid was added and the mixture vortexed, incubated for 5 minutes, and centrifuged at 13,000 x g for 3 min. The pellet was resuspended using 100 μL of double-distilled water (ddH2O), along with 2 μL of butylated hydroxytoluene (BHT) stock/BHT (100X). The final volume was adjusted to 200 μL with ddH_2_O. In each vial, 200 μL of standard or sample was combined with 600 μL of Developer VII/thiobarbituric acid (TBA) reagent. The mixture was incubated at 95°C for 60 min and cooled to room temperature in an ice bath for 10 min. 200 μL of the reaction mixtures (containing MDA-TBA adduct) were transferred into a 96-well plate and the absorbance was measured at OD 532 using a SpectraMax M2e (Molecular Devices, San Jose, CA) microplate reader; lipid peroxide level as MDA concentration (nmol/ml) was calculated as described in the kit.

**Protein Carbonyl -** For each sample, 100 μl of 2,4-dinitrophenylhydrazine (DNPH) was added and mixed before being incubated for 10 min to estimate protein carbonyl content. Then, 30 μl of Trichloroacetic Acid Solution (TCA) was added to each sample, vortexed and placed on ice for 5 minutes. The sample was spun at maximum speed for 2 min, supernatant was removed, and 500 μl of chilled acetone was added to each tube; the pellet was washed and placed at -20°C for 5 min. The samples were centrifuged for 2 min and the acetone was extracted. Following, 200 μl of guanidine solution was added, and 100 μl of each sample was transferred to a 96-well plate. The OD was measured at 375 nm using a SpectraMax M2e (Molecular Devices, San Jose, CA) microplate reader. For measurement of protein content per sample, 5 μl of each guanidine solution dissolved sample was transferred, and protein content was estimated using the BCA kit, with BSA serving as the standard protein. The protein carbonyl content (nmol/mg protein) was calculated following manufacturer's instructions.

**Superoxide Dismutase (SOD) -** Serum SOD activity was measured by generating superoxide anions through the activity of xanthine oxidase. The dismutation of the superoxide anion into H_2_O_2_ and O_2_ is catalyzed by SOD. The resulting water-soluble formazan dye, produced when superoxide anions act on water-soluble tetrazolium (WST-1), is detected by the increase in absorbance at 450 nm. The level of formazan dye produced is inversely proportional to the activity of SOD in the sample. For the assay, serum samples were added to the designated wells, followed by the addition of the WST-1 working solution and enzyme working solution, incubated for 20 minutes at 37°C, and absorbance at 450 nm was measured using a SpectraMax M2e (Molecular Devices, San Jose, CA) microplate reader; SOD activity (units/mg protein) was calculated following manufacturer’s instructions.

**Catalase (CAT) -** To measure CAT, the CAT in the samples reacted with H_2_O_2_ to generate water and oxygen. Any remaining unconverted H_2_O_2_ reacted with the OxiRed Probe, producing a product that was measured colorimetrically at OD 570 nm using a SpectraMax M2e (Molecular Devices, San Jose, CA) microplate reader. The signal obtained was inversely proportional to the CAT activity in the sample, and the activity (mU/mg protein) was calculated according to manufacturer’s instructions.

**GSH and GSSG -** The GSH+GSSG/GSH assay relies on the glutathione recycling system facilitated by DTNB and glutathione reductase. Glutathione (GSH) and DTNB undergo a reaction that results in the formation of yellow-colored 2-nitro-5-thiobenzoic acid. The concentration of GSH is determined by measuring the absorbance at 412 nm. The GSSG can be converted back to GSH through the action of glutathione reductase. Subsequently, GSH undergoes another reaction with DTNB, resulting in the production of additional 2-nitro-5-thiobenzoic acid. The presence of glutathione (GSH) was identified by excluding the glutathione reductase from the reaction mixture.

To prepare the sample, 100 μL was mixed with 25 μL of 5% 5-sulfosalicylic acid (SSA). The mixture was centrifuged at 8000 x g for 10 min at 4°C and the supernatant was used for the glutathione assay. The reaction mix contained 20 μL of NADPH generating mix, 20 μL of glutathione reductase, and 120 μL of glutathione reaction buffer. To detect the reduced form of glutathione alone, 20 μL of the glutathione reaction buffer was used instead of 20 μL of glutathione reductase. To each well, 160 μL of the reaction mix was added and incubated for 10 min, resulting in the production of NADPH. Following, 20 μL of either the GSH standard solutions or the sample solution was added, and the plate incubated for 10 min. After adding 20 μL of the substrate solution, the mixture was incubated for 10 min, and the absorbance at 415 nm was measured using a SpectraMax M2e (Molecular Devices, San Jose, CA) microplate reader. The GSH concentration was measured in the sample solutions using the standard glutathione calibration curve. A standard curve for detecting reduced glutathione using reduced glutathione, as well as a standard curve for detecting total glutathione using a total glutathione standard curve, was obtained, and GSH and GSSG contents (µg/mL) were calculated.

**Glutathione Peroxidase (GPx) –** The GPx assay was performed using ab102530 - Glutathione Peroxidase Assay (Colorimetric) Kit following the instructions provided with the kit ([www.abcam.com/ab102530](http://www.abcam.com/ab102530)). The reagents, including NADPH standard, GSH, GR, cumene hydroperoxide, and GPx positive control, were prepared per the kit instructions. The plate was prepared with a standard range of NADPH concentrations (0 nmol, 20 nmol, 40 nmol, 60 nmol, 80 nmol, and 100 nmol/ 100 μL/well) and various samples (50 μL), along with positive control (50 μL) and reagent control (50 μL) wells, all in duplicate. A reaction mix was prepared by combining the necessary components for each reaction. This included adding 33 μL of assay buffer, 3 μL of 40 mM NADPH solution, 2 μL of GR solution, and 2 μL of GSH solution. For the sample, positive control, and reagent control wells, 40 μL of reaction mix was added. The plate was incubated at room temperature for 15 minutes and then measured at an optical density of 340 nm (OD340) using SpectraMax M2e (Molecular Devices, San Jose, CA) microplate reader. For the sample, positive control, and reagent control wells, a 10 μL solution of cumene hydroperoxide was added, and output (A1) at OD340 was measured at T1. The plate was then incubated at 25ºC for 5 minutes in the dark, and the output (A2) was measured at OD340 at T2 using SpectraMax M2e (Molecular Devices, San Jose, CA) microplate reader. GPx activity was determined using the calculations provided with the kit. A unit of GPx was defined as the enzyme quantity that caused the oxidation of 1.0 μmol of NADPH to NADP+ per minute at 25°C, following the conditions of the assay kit.

**Glutathione Reductase (GR) -** In the assay, GR converts GSSG to GSH, which then reacts with 5,5′-Dithiobis (2-nitrobenzoic acid) (DTNB) to produce 5-thio-2-nitrobenzoic acid (TNB^2-^). The samples underwent treatment to eliminate GSH prior to the assay. To 100 μL of serum, 5 μL of 3% H_2_O_2_ was added, mixed, and incubated at 25°C for 5 minutes. After that, 5 μL of CAT was added, mixed, and left to incubate at 25°C for an additional 5 min. Following, 50 μL of the pre-treated samples were added to a 96-well plate. A reaction mix of 50 μl was prepared by combining GR assay buffer (40 μL), DTNB solution (2 μL), NADPH-GNERAT solution (2 μL), and GSSG solution (6 μL) and added to each test sample and mixed. The OD405 nm at time 1 (reading A1) was measured using a SpectraMax M2e (Molecular Devices, San Jose, CA) microplate reader. Subsequently, OD405nm was measured again at time 2 (reading A2), and finally, ΔA405 nm = A2 – A1, and GR activity (mU/mg protein) was calculated.
